# Live imaging of alterations in cellular morphology and organelles during cornification using an epidermal equivalent model

**DOI:** 10.1038/s41598-020-62240-3

**Published:** 2020-03-26

**Authors:** Sari Ipponjima, Yuki Umino, Masaharu Nagayama, Mitsuhiro Denda

**Affiliations:** 1 Research Center of Mathematics for Social Creativity, Research Institute for Electronic Science, Hokkaido University, Sapporo, Japan; 2 Shiseido Global Innovation Center, Yokohama, Japan

**Keywords:** Cell biology, Cell death, Organelles

## Abstract

The stratum corneum plays a crucial role in epidermal barrier function. Various changes occur in granular cells at the uppermost stratum granulosum during cornification. To understand the temporal details of this process, we visualized the cell shape and organelles of cornifying keratinocytes in a living human epidermal equivalent model. Three-dimensional time-lapse imaging with a two-photon microscope revealed that the granular cells did not simply flatten but first temporarily expanded in thickness just before flattening during cornification. Moreover, before expansion, intracellular vesicles abruptly stopped moving, and mitochondria were depolarized. When mitochondrial morphology and quantity were assessed, granular cells with fewer, mostly punctate mitochondria tended to transition to corneocytes. Several minutes after flattening, DNA leakage from the nucleus was visualized. We also observed extension of the cell-flattening time induced by the suppression of filaggrin expression. Overall, we successfully visualized the time-course of cornification, which describes temporal relationships between alterations in the transition from granular cells to corneocytes.

## Introduction

The human epidermis is a heterogeneous, multilayered structure constructed from keratinocytes^1^. All keratinocytes are originally produced at the lowest layer of the epidermis, the stratum basale (SB), following which they move towards the skin surface while differentiating through the stratum spinosum (SS) and stratum granulosum (SG) and finally transform into corneocytes at the border between the SG and the most outer layer, the stratum corneum (SC). Due to the robust hydrophobic features of the SC and tight junctions that form at the second layer of the SG, these factors play a crucial role in protection against physical damage and harmful factors outside the body as well as the prevention of water loss from inside the body^2–4^.

The terminal differentiation of the uppermost granular cells to corneocytes, the cells of the SC, is called cornification, which is a kind of programmed cell death. During cornification, a variety of phenomena occur in granular cells^3^. One of the most obvious changes is in their thickness. Corneocytes are very thin with a thickness of approximately 0.2–0.5 μm^5^. Furthermore, corneocytes do not contain nuclei and other organelles but rather are filled with densely packed keratin filaments throughout their cytoplasm^3,6^. The condensation of keratin filaments is induced by interaction with filaggrin monomers^7,8^. At the cell periphery, the cornified envelope, a structure consisting of various cross-linked intracellular proteins such as involucrin and loricrin, is formed^9^. Adjacent corneocytes are interconnected by corneodesmosomes, which are modified desmosomes, and their intercellular space is filled with lipids^2^. Several recent reports have elucidated that autophagy is involved in the elimination of nuclei and mitochondria during terminal differentiation^10,11^. It has also been reported that DNA is degraded by both DNase1L2 and DNase2^12^. Since corneocytes differ substantially from granular cells, significant and dynamic alterations should occur during cornification. However, the temporal details of this process remain unclear.

Epidermal equivalent models reconstructed by human-derived epidermal keratinocytes are widely used for many studies. The epidermal equivalent models are comparable to the native human epidermis regarding epidermal structure and its differentiation pattern^13–15^. This model enables investigation of physiological changes in keratinocytes themselves without the effects of immune systems, fibroblasts, or sensory neurons^13^. Moreover, intracellular structures in this model can be labelled simply using fluorescent probes, and this model can be stimulated with external inducers or inhibitors and is also appropriate for knockdown experiments with small interfering RNA (siRNA)^16–19^. Taking advantage of these features, in this study, we performed three-dimensional time-lapse imaging of a human epidermal equivalent model with two-photon microscopy focusing on cellular morphology, acidic vesicles, and mitochondria to determine what happens during cornification and what is the order of these events. Additionally, we demonstrated that the knockdown of filaggrin in the epidermal model increased the duration of cornification.

## Results

### Morphological changes in granular cells during cornification consisted of not only flattening but also expansion

**Figure 1:**
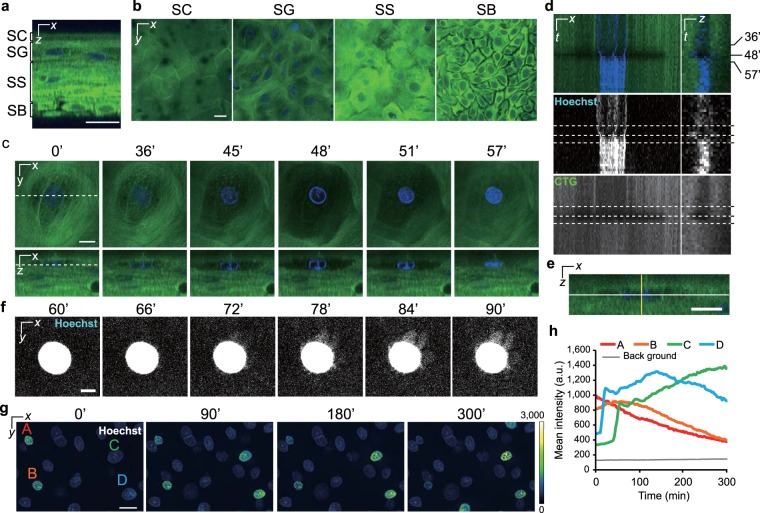
Alterations in the cellular and nuclear morphology during cornification. (**a**) A representative orthogonal image of an epidermal model labelled with Hoechst 33258 (blue) and CTG (green). (**b**) A series of optical sectioned images of the SC, SG, SS, and SB. (**c**) Time-lapse images collected with a 3-min interval. Dashed lines indicate the sectional planes. The granular cell shape began to change at 36 min, following which the cell expanded until 48 min and became flattened at 57 min. (**d**) Kymographs of the nucleus and cell shape taken from time-lapse images in **c**. Kymographs on the left and right were generated from the white and yellow lines in **e**, respectively. The upper, middle, and lower panels show the merged images, nuclear signals, and CTG signals, respectively. Dashed lines show the time at 36, 48, and 57 min. (**e**) A cropped orthogonal image at 0 min in **c**. (**f**) Maximum intensity projections of a nucleus after flattening. These images consist of 10 images in the *z*-direction (6 μm). Note that the brightness was adjusted to visualize low signals. (**g**) Pseudocoloured maximum intensity projections of nuclei before and after flattening. These images consist of 20 images in the *z*-direction (12 μm). The A and B cells were already flattened before observation. The C and D cells became flattened during observation. (**h**) A graph of the nuclear mean intensities of the marked cells (A–D) in **g**. Scale bars, 20 μm (**a**,**b**,**g**), 10 μm (**c**,**e**), or 5 μm (**f**).

The epidermal equivalent model was labelled with CellTracker Green (CTG) and Hoechst 33258 for the visualization of cell shape and nucleus, respectively. Similar to the native human epidermis, the epidermal model formed squamous epithelium (Fig. 1a,b, Supplementary Movie 1)^20^. We then collected three-dimensional time-lapse images with a 3-min interval over 3–5 hours and successfully captured morphological changes in granular cells during cornification. Surprisingly, the captured images revealed that the granular cells and their nuclei temporarily expanded in the *z*-direction just before flattening (Fig. 1c–e, Supplementary Movie 2). During that time, the CTG signals gradually decreased. Subsequently, the cells became flattened, and an increase in the Hoechst 33258 and CTG signals was observed. The nuclei shrank in the *xy*-plane during the period of morphological change. Interestingly, several minutes after the cells flattened, we noticed slight radial Hoechst 33258 signals that leaked from the nuclear region in most of the cells (14 out of 17 cells) (Fig. 1f, Supplementary Movie 3). Thereafter, the intensity of the Hoechst 33258 signal slowly and gradually decreased (Fig. 1g,h, Supplementary Movie 4). This decrease is thought to reflect the degradation of nuclear DNA, as reported previously^21,22^. Although collapse of the nuclear lamina should be required for DNases to access DNA, when the nuclear lamina deteriorates had been unclear^12,23^. Our results suggest that the nuclear lamina collapses at least several minutes after flattening.

### Acidic vesicles in granular cells suddenly stopped moving just before morphological changes were observed

**Figure 2:**
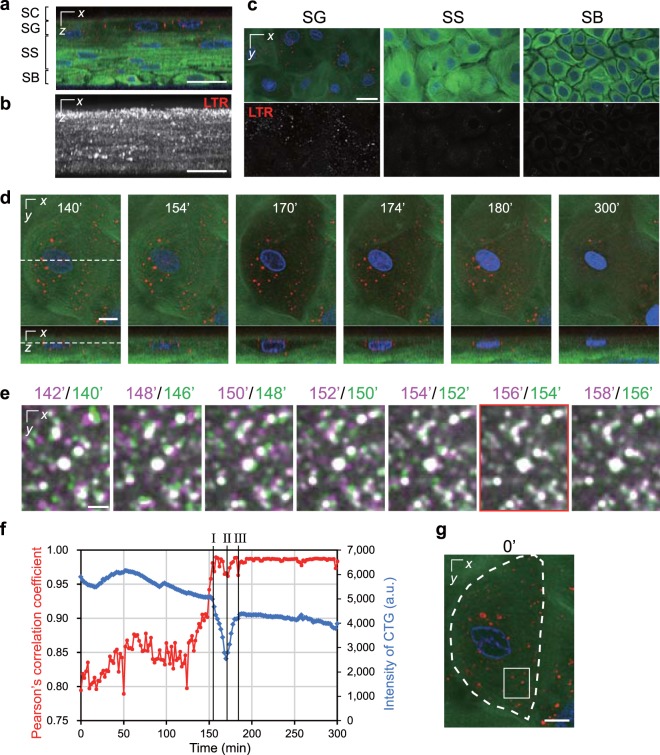
The temporal relationship between the stoppage of acidic vesicles and morphological change. (**a**) A representative orthogonal image of the epidermal model labelled with Hoechst 33258 (blue), CTG (green), and LTR (red). (**b**) A maximum intensity projection of only LTR signals in **a**. (**c**) A series of optical sectioned images of the SG, SS, and SB. The upper and lower panels show the merged images and LTR signals, respectively. (**d**) Time-lapse images collected with a 2-min interval. Dashed lines indicate the sectional planes. The granular cell shape began to change at 154 min, following which the cell expanded until 170 min and became flattened at 180 min. (**e**) Enlarged merged images of LTR signals from the neighbouring timeframe taken from time-lapse images. Magenta indicates 2 min after the signals in green. (**f**) A graph of Pearson’s correlation coefficient for the colocalization of two LTR signals versus time (red line) and intensity of CTG (blue line) that were obtained from the time-lapse images. The vertical lines I, II, and III indicate the beginning of the morphological change, cellular expansion, and complete flattening, respectively. (**g**) The regions for analysis in **e** (the white rectangle) and **f** (indicated by a dashed line). The data in **e**–**g** based on the time-lapse images in **d**. The data are representative of 22 cells (4 independent experiments). Scale bars, 25 μm (**a**–**c**), 10 μm (**d**,**g**), or 2.5 μm (**e**).

Next, we utilized LysoTracker Red (LTR) to label acidic organelles, including lysosomes, in addition to Hoechst 33258 and CTG. Acidic vesicles with highly intense LTR signals were abundant in the SG but rare in the SS and SB (Fig. 2a–c). LTR signals at the SC were scarcely detected. In time-lapse images collected at a 2-min interval, most acidic vesicles in the granular cells before they exhibited a morphological change moved so quickly that they were captured at different positions in neighbouring frames. However, the vesicles suddenly stopped moving around the time when the cell started to expand (Fig. 2d–g, Supplementary Movie 5). The vesicles stopped in all cells that became flattened during observation. After flattening, the LTR signals gradually decreased and then mostly disappeared (Fig. 2d, Supplementary Movie 5). This probably means that the LTR diffused due to changes in the membrane permeability of the vesicles or because their internal pH increased^24^. Additionally, the average duration between each event in uppermost granular cells that became flattened was measured in the experiments with this label (*n* = 22). As a result, the vesicles stopped moving essentially within a few minutes (2.3 ± 3.1 min) before the morphological changes began. The average time from the beginning of the morphological change to maximum cell expansion was 9.2 ± 3.3 min. The average time from the maximum cell expansion to flattening was 9.0 ± 3.0 min. The average time from flattening to the detection of DNA leakage was 3.9 ± 4.7 min (detected in 21 out of 22 cells).

### Alteration in the mitochondrial condition during cornification

**Figure 3:**
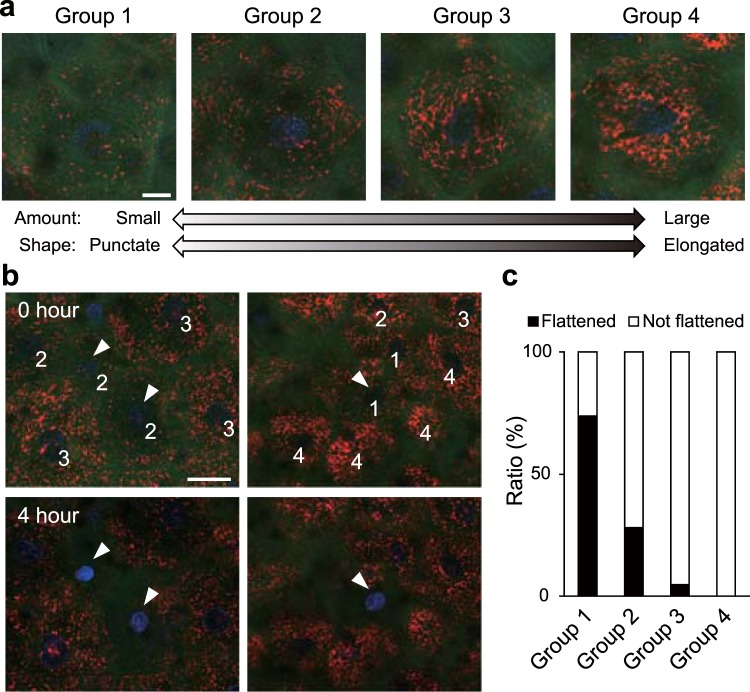
Alterations in mitochondrial condition during cornification. (**a**) Representative images of granular cells just below corneocytes in each group. The epidermal model was labelled with Hoechst 33258 (blue), CTG (green), and TMRE (red). Most mitochondria in group 1 had a punctate structure. The granular cells in group 2 had few mitochondria with a punctate and elongated structure. The mitochondrial structures in group 4 were comparable to those in the lower layer, while the granular cells in group 3 had fewer mitochondria than those in group 4. (**b**) Representative images at the start of observation (upper images) and four hours later (lower images). The numbers on the cells shows their group categorization. The arrowheads indicate flattened cells after four hours. (**c**) A graph of the proportion of cells that had flattened and cells that had not flattened after four hours. The data are representative of 483 cells (8 independent experiments). The ratios of the number of flattened and not flattened cells were 62:22 (group 1), 23:59 (group 2), 6:120 (group 3), and 0:191 (group 4). Scale bars, 10 μm (**a**) or 25 μm (**b**).

To visualize mitochondria, we initially utilized tetramethylrhodamine ethyl ester (TMRE), which accumulates in polarized mitochondria. Live imaging of the epidermal model labelled by Hoechst 33258, CTG, and TMRE revealed that some granular cells that contacted corneocytes had punctate mitochondria, unlike most other keratinocytes, which had a large amount of elongated mitochondria (Supplementary Fig. 1). To investigate the relationship between mitochondrial conditions and cornification, we categorized granular cells just below corneocytes into the following four groups: (1) cells with a small amount of mitochondria with only punctate structures, (2) cells with a small amount of mitochondria with both punctate and elongated structures, (3) cells with a moderate amount of slightly fragmented mitochondria, (4) cells with a large amount of elongated mitochondria (similar to those in lower granular cells) (Fig. 3a). Then, each granular cell was categorized depending on whether it became flattened within four hours (Fig. 3b). A total of 74% of the cells in group 1 became flattened, which was the highest percentage observed among all groups; the percentage of cells that became flattened was decreased in order from group 1 to group 4 (Fig. 3c). These results suggest that the cells with fewer and more punctate mitochondria tend to undergo morphological change.

**Figure 4:**
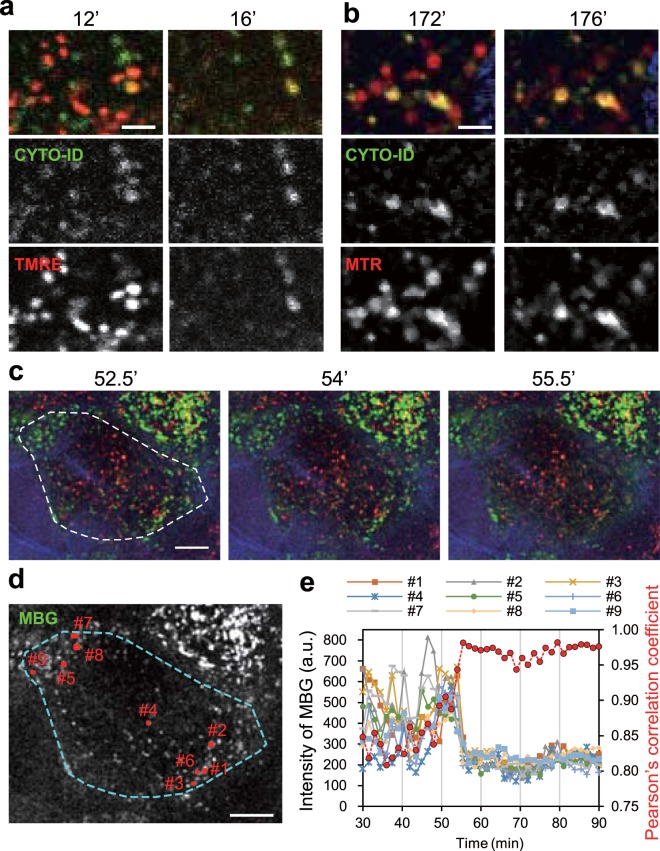
The spatiotemporal relationships among mitochondria, autophagosomes, and acidic vesicles. (**a**) Representative enlarged time-lapse images of granular cells labelled with Hoechst 33258 (blue), CYTO-ID (green), and TMRE (red). The upper, middle, and lower panels show merged images, CYTO-ID signals, and TMRE signals, respectively. The data are representative of 17 cells (4 experiments). (**b**) Representative enlarged time-lapse images of granular cells labelled with Hoechst 33258 (blue), CYTO-ID (green), and MTR (red). The upper, middle, and lower panels show merged images, CYTO-ID signals, and MTR signals, respectively. The data are representative of 32 cells (4 experiments). (**c**) Representative time-lapse images of granular cells labelled with CTB (blue), MBG (green), and LTR (red). A dashed line indicates the region for analysis of Pearson’s correlation coefficient in **e**. (**d**) An image of MBG signals in **c** at 52.5 min before the disappearance of mitochondrial signals. Small red rectangles show the mitochondrial regions analysed in **e**. (**e**) A graph of the intensity of MBG signals in each region of the rectangles (#1–9) in **d** and Pearson’s correlation coefficient for the colocalization of LTR signals versus time (red dashed line). The data presented in **c**–**e** are representative of 19 cells (5 experiments). Scale bar, 2.5 μm (**a**,**b**) or 10 μm (**c**,**d**).

Since a previous study demonstrated that autophagy contributes to the degradation of mitochondria in the SG, we next utilized CYTO-ID Green to label autophagosomes in addition to TMRE or MitoTracker Red (MTR) to label mitochondria and Hoechst 33258 to label nuclei^11^. Autophagosomes were abundant in the SG and partly colocalized with the mitochondrial signal (Supplementary Figs. 2 and 3). We also performed time-lapse imaging with a 2-min interval, which showed that all TMRE signals that did not colocalize with autophagosomes rapidly disappeared when the cell began to undergo morphological changes (Fig. 4a, Supplementary Movie 6). In the case of the MTR signals, they decreased but did not completely disappear (Fig. 4b, Supplementary Movie 7). These results probably differ because MTR tends to bind proteins within mitochondria (see Methods and Supplementary Fig. 7). Accordingly, the disappearance of TMRE signals before flattening indicates mitochondrial depolarization rather than degradation. To clarify the temporal relationship between mitochondrial depolarization and the stoppage of acidic vesicles, we carried out live imaging using MitoBright Green (MBG) to visualize mitochondria together with LTR and CellTracker Blue (CTB). Time-lapse images with a 1.5-min interval showed the remaining mitochondrial signals disappeared as the acidic vesicles stopped moving (Fig. 4c–e, Supplementary Movie 8). Taken together, our results and those of previous studies suggest that the mitochondria in the uppermost granular cells may become fragmented and degraded by autophagy, after which the surviving mitochondria finally depolarize while intracellular vesicles stop moving just before the cell shape changes.

### Filaggrin deficiency increased the period of flattening

Filaggrin is expressed as the very large precursor protein profilaggrin, a major component of keratohyalin granules^8,25–27^. During terminal differentiation, profilaggrin is dephosphorylated and proteolysed into multiple filaggrin monomers that directly bind and condense keratin filaments^6,7,28–33^. To determine whether filaggrin is involved in the morphological change observed during cornification, we generated a filaggrin knockdown (FLG-KD) epidermal model using siRNA. Filaggrin deficiency was confirmed by quantitative PCR analysis and immunostaining (Supplementary Fig. 4a,b). Histological sections of the FLG-KD model showed few keratohyalin granules and the sparse presence of nuclear remnants in SC (Supplementary Fig. 4c). Meanwhile, the ultrastructure of the SC in FLG-KD models showed abnormal corneodesmosomes and decreased electron density compared to control models (Supplementary Fig. 4d). Three-dimensional immunostaining revealed abnormally distributed corneodesmosin (CDSN, a component of the corneodesmosome) throughout the cytoplasm of the uppermost cells of the FLG-KD model, whereas CDSN in control models was localized at the cell periphery (Supplementary Fig. 4e). We also assessed the effect of filaggrin deficiency on the expression of differentiation markers and found that filaggrin2 (FLG2) and loricrin (LOR) expression was significantly reduced, whereas CDSN and ABCA12 expression was hardly changed (Supplementary Fig. 4f). Altogether, these results indicated that epidermal differentiation in the FLG-KD model was impaired, which was consistent with a previous report^16,34^.

**Figure 5:**
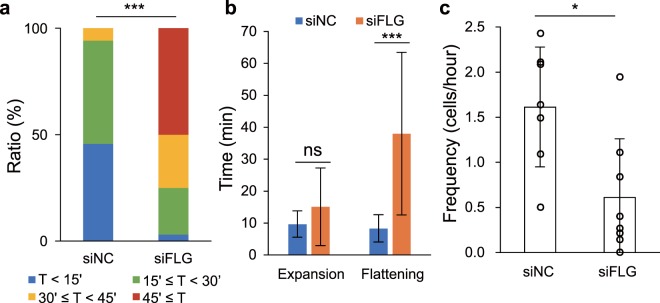
Analysis of live imaging data from the FLG-KD epidermal model. (**a**) A graph of the time taken for morphological change to occur in the control (siNC) (*n* = 35) and FLG-KD models (siFLG) (*n* = 32). Wilcoxon rank sum test: *p* = 1.5 × 10^−10^. (**b**) A graph of the average times from the start of morphological change to expansion (shown as “Expansion”) and from expansion to flattening (shown as “Flattening”) in the control (*n* = 28) and FLG-KD (*n* = 22) models. The cells that underwent a morphological change, expansion, and flattening during observation were counted. Wilcoxon rank sum test: *p* = 0.18 (Expansion), *p* = 2.2 × 10^−12^ (Flattening). Details are shown in Supplementary Fig. 6. (**c**) A graph of the number of cells that began to undergo a morphological change per hour in the control and FLG-KD models (7 (control) and 8 (FLG-KD) independent experiments). Wilcoxon rank sum test: *p* = 0.014. *p* values, *<0.05, **<0.01, ***<0.001.

We subsequently carried out live imaging using FLG-KD models labelled with a combination of Hoechst 33258, CTG, and LTR (Supplementary Movies 9 and 10). We found that the duration of the morphological change in the FLG-KD models was much longer than that in control models (Fig. 5a, Supplementary Fig. 5, Supplementary Movie 11). A detailed analysis showed that the time from maximum expansion to flattening was significantly longer in the FLG-KD models than in control models, while the time from the beginning of the morphological change to expansion was not significantly different (Fig. 5b, Supplementary Fig. 6). In contrast, there was no difference in the order of the events observed during cornification, i.e., the stoppage of acidic vesicles as a first step, subsequent expansion of the cell, and eventual flattening of the cell, between finally flattened cells in FLG-KD models and those in control models. Meanwhile, the proportion of the cells beginning to undergo the morphological change in the FLG-KD models was significantly lower than that in the control models (Fig. 5c). Altogether, these results suggest that keratin condensation by filaggrin occurs mainly after cell expansion and accelerates cell flattening, which may lead to appropriate cornification.

## Discussion

In this study, we succeeded in visualizing changes in the cell shape and organelles during terminal differentiation using a living human epidermal equivalent model. Based on many previous studies, an elevated cytoplasmic calcium ion ([Ca^2+^]_i_) concentration has been thought to be a key factor for triggering cornification^35–37^. Indeed, a recent study using intravital mouse imaging demonstrated that the [Ca^2+^]_i_ was transiently elevated approximately 40 min before cornification^38^. Therefore, the series of morphological changes we observed can be estimated as a result of the elevation of [Ca^2+^]_i_. Interestingly, we found that acidic vesicles stopped moving simultaneously with mitochondrial depolarization just before the shapes of the cell and nucleus changed. This result suggests that some common intracellular structure or molecule intervene between the elevation in [Ca^2+^]_i_ and these phenomena. As the cytoskeleton is a structure involved in organizing cell shape, it would be related to these phenomena. Several reports have shown that a cortical actin pattern is formed in differentiated keratinocytes^39,40^. Actin filaments are also known to be responsible for distributing organelles and mitochondrial depolarization^41–43^. Moreover, Gutowska-Owsiak *et al*. showed that actin formed structures associated with filaggrin-containing keratohyalin granules and suggested that actin disruption accelerates keratinocyte differentiation programmes by filaggrin release from keratohyalin granules^44^. Therefore, the series of morphological changes we observed may result from actin cytoskeleton collapse. Microtubules might also be involved in these phenomena because a previous study indicated that microtubules were required for the formation of corneocytes^45^. To elucidate details of the molecular mechanism by which the morphological change is induced, a combination study with a molecular biological approach and time-lapse imaging would be valuable.

The capability of each granular cell in the uppermost layer of the SG to cornify depended on the degree of its mitochondrial fragmentation and degradation, as shown in our results in Fig. 3, suggesting that mitochondrial conditions play a role in preparing for the induction of flattening. Generally, mitochondrial morphology is closely associated with the ability of mitochondria to produce energy, and the balance between mitochondrial fission and fusion is important for the maintenance of mitochondrial quantity and quality^46,47^. Mitochondrial fusion is promoted in cells under conditions of high energy demand, whereas mitochondrial fission and subsequent degradation by autophagy are induced in cells under conditions of low energy demand^48^. Thus, mitochondrial fragmentation in granular cells just below corneocytes might result from reduced energy demand in the cells. In fact, it has been reported that transcription levels in keratinocytes were markedly decreased in SG compared with SB and SS^49^. Meanwhile, various intracellular and environmental factors, including reactive oxygen species (ROS) and endoplasmic reticulum (ER) stress, can disturb mitochondrial quality^50,51^. If mitochondria are injured by these factors, the resultant dysfunctional mitochondria are fragmented and subsequently degraded by autophagy to maintain healthy mitochondrial networks^52^. Moreover, severe mitochondrial damage leads to excessive mitochondrial fission and cell death, such as apoptosis, necrosis, and autophagic cell death^47,51,53,54^. Several reports have indicated the involvement of autophagy in keratinocyte differentiation^10,11^. Reports using an epidermal equivalent model by Moriyama *et al*. demonstrated that BNIP3 in granular cells induced autophagy and was involved in the terminal differentiation and maintenance of the epidermis^11^. In our study, autophagosomes were certainly colocalized with mitochondria. This finding supports the notion that mitochondria are partly degraded by autophagy in granular cells before the start of morphological change. It is debatable, however, whether autophagy itself is essential for cornification in the physiological epidermis^10,55–61^. Factors that influence mitochondrial conditions, such as ROS and ER stress, may participate in preparing for cornification. Indeed, several previous studies showed the importance of ROS and ER stress in keratinocyte differentiation^61–64^.

Since filaggrin monomers directly bind and condense keratin filaments, keratin bundling by filaggrin monomers could provide the force required for flattening, at least in part^6^. Therefore, it is reasonable to consider that the extension of the flattening time in the FLG-KD model resulted from abnormal keratin condensation. Meanwhile, the frequency of flattening in the FLG-KD model was decreased in our study. One possibility for this finding is that the extended duration of the morphological change in the FLG-KD model affected the frequency of flattening. Only the uppermost granular cells underwent the morphological change, and the granular cells just below the transforming cells did not show changes in morphology in our controls or the FLG-KD models. These cells might not be able to undergo cornification until the cells above them are completely flattened. Thus, the elongated time frame of the observed morphological change would delay the beginning of cornification, decreasing the frequency of transformation. On the other hand, it should be noted that the expression of some differentiation markers was significantly decreased in FLG-KD models. This result is consistent with previous studies, although why filaggrin knockdown affects their expression remains unknown^16,34^. Because the series of events during cornification in normal models also occurred in the FLG-KD models, the impairment of differentiation in our FLG-KD models might have only a slight effect on the frequency of flattening.

**Figure 6:**
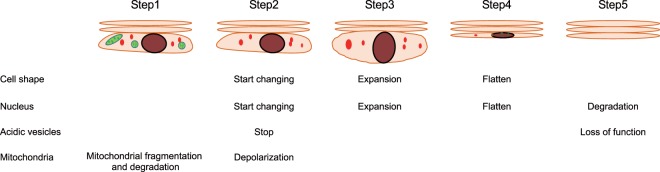
Summary of the scheme proposed in our study. First, mitochondria undergo fission, and fragmented mitochondria in the SG are degraded by autophagy (Step 1). After the stoppage of acidic vesicles coinciding with mitochondrial depolarization, granular cells soon begin to change their cellular and nuclear morphology (Step 2). Next, the cell and nucleus expand for approximately 10 min (Step 3) and then becomes flattened over approximately 10 min (Step 4). Then, functions of acidic vesicles may be lost, and DNA is gradually degraded (Step 5).

The events we observed in cornifying cells are summarized in Fig. 6. Mitochondria in granular cells just under corneocytes are gradually fragmented and degraded by autophagy. Because the morphology of granular cells with fewer and more punctate mitochondria tended to begin changing, the deterioration of mitochondria may be a sign to prepare to proceed to final transformation. Just before the granular cell undergo a morphological change, rapid intracellular acidic vesicles suddenly stop moving, and the remaining mitochondria simultaneously depolarize. Subsequently, the cell expands vertically for approximately 10 min. After reaching its maximum size, the cell completely flattens over the same length of time taken for the cell to expand to its maximum size. Filaggrin is involved in cell flattening after expansion rather than cell expansion. Although DNA remains in the cell just after it has flattened, a portion of the DNA leaks from the nucleus within several minutes, possibly due to collapse of the nuclear membrane. Then, as reported, the DNA is slowly degraded probably by DNase1L2 and DNase2 and eventually lost over a half a day^12^.

In the present study, we focused on sequential changes in not only the cell shape but also organelles during cornification. However, the process of cornification also includes the other phenomena, i.e., the formation of a cornified envelope, elevation of the [Ca^2+^]_i_, and secretion of intercellular lipids. Details of the relationships between our results and these phenomena remain poorly understood. Investigations of the relationships among these phenomena will lead to a further understanding of cornification.

## Methods

### Preparation of epidermal equivalent models

Normal human epidermal keratinocytes (NHEKs) from Kurabo (Osaka, Japan) were cultured in EpiLife-KG2 (Kurabo) containing 0.06 mM Ca^2+^ and passaged three times. Millicell hanging cell culture inserts with a polyethylene terephthalate membrane pore size of 0.4 µm (Millipore, Billerica, MA) were precoated with CELLstart (Thermo Fisher Scientific, Waltham, MA) in 1:50 diluted Dulbecco’s phosphate-buffered saline (PBS). A total of 2.3 × 10^5^ keratinocytes suspended in 500 μl of CnT-Prime medium (CELLnTEC, Berne, Switzerland) were plated on each insert (defined as day 0). One millilitre of CnT-Prime medium was added to the outside of the insert. At day 3, the medium both inside and outside the inserts was switched to CnT-PR-3D differentiation medium (CELLnTEC) containing 50 μg/mL ascorbic acid. Cultures were submerged in differentiation medium overnight and then lifted to the air-medium interface by removing excess medium from the insert and reducing the volume of differentiation medium on the outside of the insert to 500 µl. Cultures were fed daily with 500 µl of the differentiation medium. Epidermal models at days 11–13 were used for experiments.

### Fluorescent labelling of epidermal equivalent models

Epidermal models were labelled with the following fluorescent dyes. Hoechst 33258 (0.2 μg/mL, Sigma-Aldrich, St. Louis, MO) was used for nuclear staining. CTB (15 μM, Thermo Fisher Scientific) and CTG (15 μM, Thermo Fisher Scientific) were used to visualize cell shape. LTR DND-99 (0.2 or 0.4 μM, Thermo Fisher Scientific) was used for acidic vesicle staining. To visualize autophagosomes, CYTO-ID Green fluorescent dye (3/1000, Enzo Life Sciences, Farmingdale, NY) was used. To visualize mitochondria, three fluorescent lipophilic cationic dyes that accumulate in polarized mitochondria were utilized: TMRE (0.1 μM, Thermo Fisher Scientific), MTR (2.0 μM, Thermo Fisher Scientific), and MBG (2.0 μM, Dojindo Molecular Technologies, Tokyo, Japan). TMRE was used as a reversible probe depending on the mitochondrial membrane potential. MTR and MBG were used to confirm mitochondrial structure because they tend to be retained in mitochondria even after a reduction in the mitochondrial membrane potential due to their ability to covalently link proteins^65^. These features were confirmed by the induction of mitochondrial depolarization in keratinocytes cultured in a monolayer (Supplementary Fig. 7). For staining with LTR DND-99, epidermal models were incubated for 1–2 hours. For staining with the other fluorescent probes, epidermal models were incubated overnight.

### Two-photon microscopy

Imaging was performed using an A1RMP microscope equipped with four gallium arsenide phosphide non-descanned detectors (Nikon, Tokyo, Japan). An N40XLWD-NIR objective lens (Nikon) was attached to the inverted microscope. The excitation wavelength at 880 nm was used in the experiments with CYTO-ID. In the other experiments, the excitation wavelength at 820 nm was used. Hoechst 33258 and CTB signals were detected using a 495-nm long-pass dichroic mirror and a bandpass barrier filter 450/50 before the 1st detector. The CTG, MBG, CYTO-ID, and Alexa Fluor 488 signals were detected using a 560-nm long-pass dichroic mirror and a bandpass barrier filter 525/50 before the 2nd detector. TMRE signals were detected by reflecting a 593-nm long-pass dichroic mirror and passing through a bandpass barrier filter 575/25 before the 3rd detector. The LTR, MTR, and Alexa Fluor 594 signals were detected by passing through a 593-nm long-pass dichroic mirror and a bandpass barrier filter 629/56 before the 4th detector. The *z*-interval was set at 0.6 μm. For live imaging, an epidermal model was incubated at 37 °C without CO_2_ regulation and humidified in an incubator chamber (INU-TIZB-NB, Tokai Hit, Shizuoka, Japan) on a piezo stage driven by a Nano-Drive (Mad City Labs, Madison, WI) controller.

### Preparation of FLG-KD models

Silencer Select Pre-Designed siRNA (s5265, Thermo Fisher Scientific) was used for filaggrin knockdown, and Silencer Select Negative Control No. 1 (Thermo Fisher Scientific) was used as a negative control. On the day before seeding onto 12-well Millicells, keratinocytes were transfected with 20 nM negative control (siNC) or FLG siRNA (siFLG) using Lipofectamine RNAiMAX (Thermo Fisher Scientific) in OptiMem (Thermo Fisher Scientific). After transfection, the cells were cultured normally and used on day 11.

### Quantitative PCR analysis

Total RNA from human keratinocytes was isolated using Isogen (Nippon Gene, Tokyo, Japan). Complementary DNA (cDNA) synthesis from 1.7 μg of total RNA was performed using SuperScript VILO Master Mix (Thermo Fisher Scientific). PCR was performed using LightCycler 480 Probes Master (Roche, Basal, Switzerland), cDNA, the appropriate probes, and specific primer pairs on a LightCycler 480 System II (Roche). The primers and probes were designed using the ProbeFinder software (Roche), freely available at the Universal ProbeLibrary Assay Design Center (https://lifescience.roche.com/global_en/brands/universal-probe-library.html) and are listed in Supplementary Table S1. Relative levels of gene expression were calculated using cDNA prepared from normal epidermal models with the ΔΔ cycle threshold method^66,67^. Glyceraldehyde-3-phosphate dehydrogenase (GAPDH) served as a reference gene.

### Histology

For haematoxylin and eosin staining, the epidermal models were fixed in 4% formaldehyde in PBS at 4 °C. The samples were embedded in paraffin and sectioned at 3 μm. The sections were photographed using a microscope (BX51 and DP74, Olympus, Tokyo, Japan) using cellSens software (Olympus).

### Immunofluorescence staining for filaggrin

For immunostaining, samples were embedded in OCT compound (Sakura Finetek, Tokyo, Japan) without fixation and frozen at −80 °C. The frozen tissues were cut to 6-μm thickness using a Microm HM550 cryostat (Thermo Fisher Scientific). The sections were fixed in 4% formaldehyde in PBS for 30 min, washed with PBS, and treated with 3% bovine serum albumin (BSA) and 0.3% Triton X-100 in PBS for 30 min at room temperature (RT). Subsequently, the sections were incubated with primary antibodies (rabbit polyclonal anti-filaggrin (1/200, H-300, Santa Cruz Biotechnology, Dallas, TX) recognizing the N-terminal region and mouse monoclonal anti-filaggrin (1/200, AKH1, Santa Cruz Biotechnology) recognizing the filaggrin monomer) overnight at 4 °C. After washing, the sections were incubated with secondary antibodies (donkey anti-mouse Alexa Fluor 488 (1/400, Thermo Fisher Scientific) and donkey anti-rabbit Alexa Fluor 594 (1/400, Thermo Fisher Scientific)) and Hoechst 33258 (0.4 μg/mL, Sigma-Aldrich). The immunostained sections were photographed using a fluorescence microscope (BX51 and DP80, Olympus) using cellSens software (Olympus).

### Three-dimensional immunofluorescence staining

Three-dimensional immunostaining was examined based on the previous study^40^. Epidermal models with membranes were detached from their inserts. The cut epidermal models were fixed in 4% formaldehyde in PBS on ice for 10 min and washed with PBS. The fixed epidermal models were permeabilized with 0.5% Triton X-100/PBS for an hour and 3% BSA in PBS for 30 min at RT. Primary and secondary antibodies were diluted in antibody diluent solution (Dako, Santa Clara, CA). The epidermal models were incubated with the primary antibodies mouse monoclonal anti-ZO-1/TJP1 antibody (1/200, ZO1–1A12, Invitrogen, Carlsbad, CA) and rabbit polyclonal anti-CDSN (1/200, #NBP2-47501, Novus Biologicals, Centennial, CO) over three nights at 4 °C on a shaker. After washing, the epidermal models were incubated with the secondary antibodies donkey anti-mouse Alexa Fluor 488 (1/400) and donkey anti-rabbit Alexa Fluor 594 (1/400) and Hoechst 33258 (0.4 μg/mL) overnight at 4 °C on a shaker. The immunostained samples were observed using a two-photon microscope with an excitation wavelength of 820 nm.

### Transmission electron microscopy

Epidermal model samples for electron microscopy were minced and fixed overnight in modified Karnovsky’s fixative. Subsequent osmium tetroxide fixation and image acquisition were carried out by a contract service (Hanaichi UltraStructure Research Institute, Okazaki, Japan).

### Analysis of mitochondrial depolarization in cultured living keratinocytes

NHEKs were plated on a 35-mm glass-bottomed dish in EpiLife-KG2 containing 0.06 mM Ca^2+^. Keratinocytes were stained with TMRE (50 nM), MBG (200 nM), or MTR (200 nM) for 20–30 min. To induce mitochondrial depolarization, a mixed solution of antimycin A (10 μM) and oligomycin complex (2 μg/mL) was added to the dish. Images were detected using the two-photon microscope as described above.

### Software and data analysis

NIS-Elements software (Nikon) was used for data analysis. All time-lapse images were processed using Imaris software (Oxford Instruments, Oxfordshire, UK) to correct slight drift during image acquisition. For noise removal, median and/or smoothing filter was used. Pearson’s correlation coefficient was obtained after correction of slight drift in the *xy*-plane using NIS-Elements software. Movies were created using NIS-Elements and Fiji software (https://fiji.sc).

### Statistics

Data are represented as the mean ± SD of at least three samples. The statistical significance of differences between the control and FLG-KD models was assessed using the Wilcoxon rank sum test. Differences with a value of *p* < 0.05 were considered statistically significant. The statistical analyses were carried out with R software (version 3.4.0 for Windows; https://www.r-project.org/).

## Supplmentary information

Supplmentary information.
Supplmentary Movie 1.
Supplmentary Movie 2.
Supplmentary Movie 3.
Supplmentary Movie 4.
Supplmentary Movie 5.
Supplmentary Movie 6.
Supplmentary Movie 7.
Supplmentary Movie 8.
Supplmentary Movie 9.
Supplmentary Movie 10.
Supplmentary Movie 11.
