# Supplementary material for: Live imaging of alterations in cellular morphology and organelles during cornification using an epidermal equivalent model
Source: Sci Rep. 2020 Mar 26;10:5515. doi: 10.1038/s41598-020-62240-3 (PMC7099034; doi:10.1038/s41598-020-62240-3)
Supplement: Supplementary file 1 — Supplmentary information. [file 41598_2020_62240_MOESM1_ESM.pdf]

Supplementary Information

Live imaging of alterations in cellular morphology and organelles during cornification using an epidermal equivalent model

Sari Ipponjima, Yuki Umino, Masaharu Nagayama, Mitsuhiro Denda

Supplementary Figure 1

Supplementary Figure 2

Supplementary Figure 3

Supplementary Figure 4

Supplementary Figure 5

Supplementary Figure 6

Supplementary Figure 7

Supplementary Table 1

Supplementary Movie legends

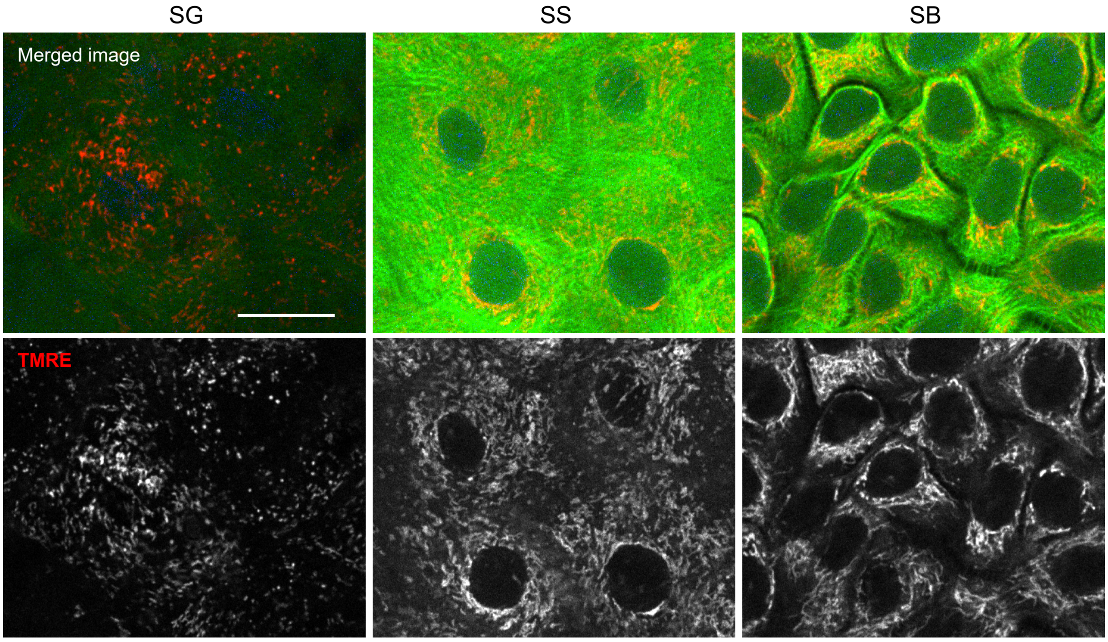

Supplementary Figure 1
Representative images of mitochondria in the SG, SS, and SB of the epidermal model labelled with Hoechst 33258 (blue), CTG (green), and TMRE (red). The upper panels show merged images, and the lower panels show only TMRE signals. Scale bar, 20 µm.

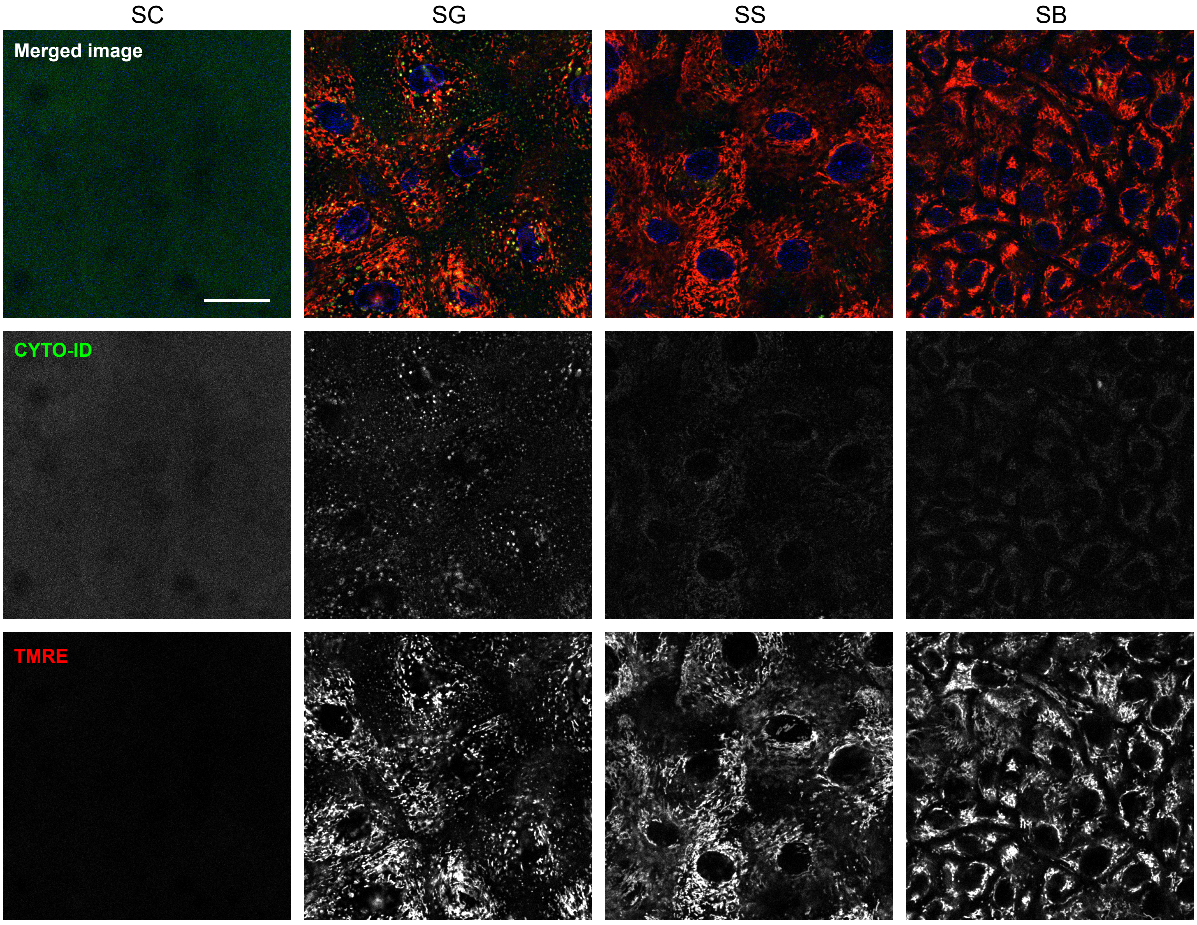

Supplementary Figure 2
A series of optical sectioned images of the epidermal model labelled with Hoechst 33258 (blue), CYTO-ID (green), and TMRE (red). The upper, middle, and lower panels show merged images, CYTO-ID signals, and TMRE signals, respectively. Scale bar, 25 µm.

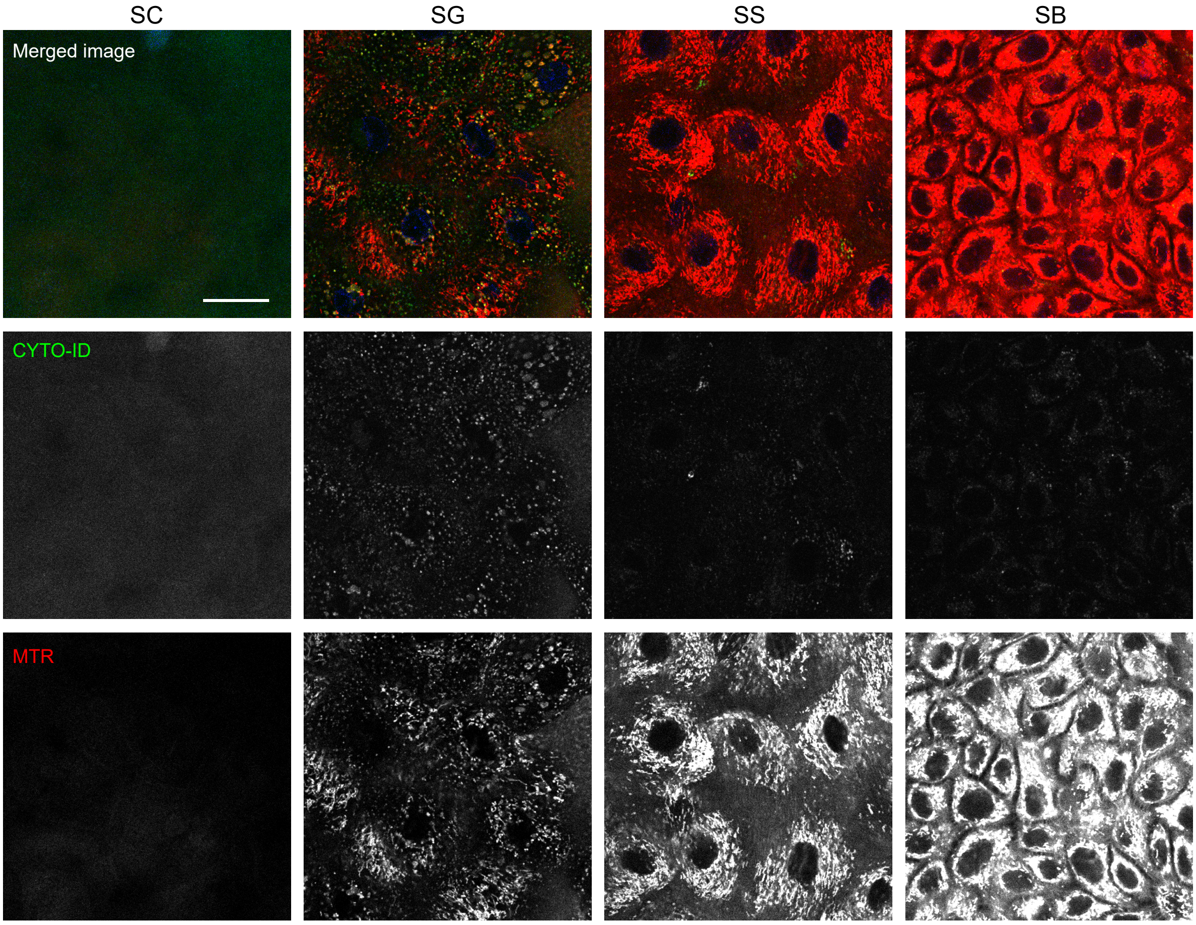

Supplementary Figure 3
A series of optical sectioned images of the epidermal model labelled with Hoechst 33258 (blue), CYTO-ID (green), and MTR (red). The upper, middle, and lower panels show merged images, CYTO-ID signals, and MTR signals, respectively. Scale bar, 25 µm.

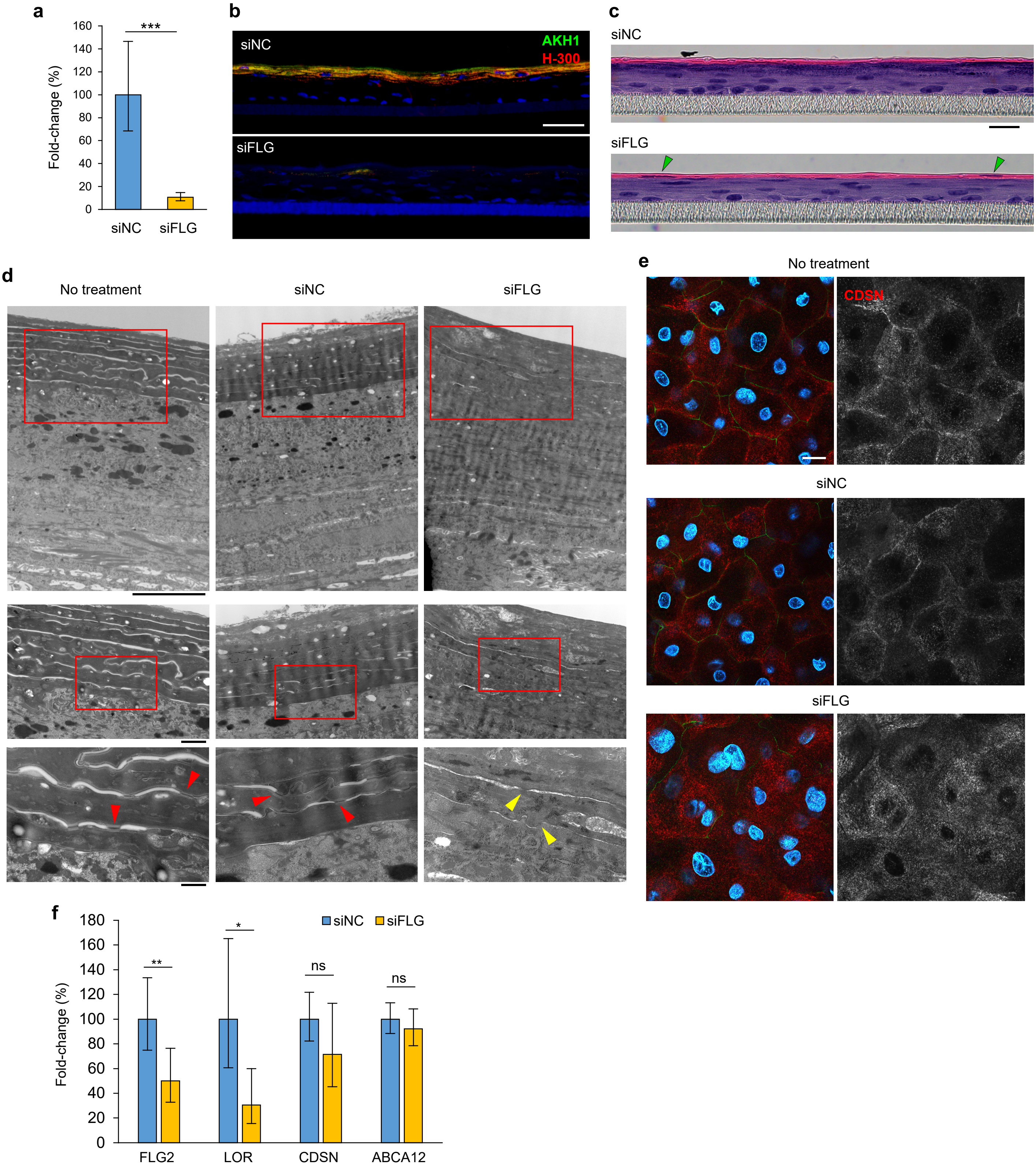

Supplementary Figure 4

Characteristics of FLG-KD models. **a**, Quantitative PCR analysis of FLG expression in control and FLG-KD models. The mRNA levels of FLG corresponding to the control were set to 100. All the experimental points were obtained from 8 independent experiments. Wilcoxon rank sum test: $p = 0.00015$. **b**, Representative immunostaining images with AKH1 (against the filaggrin monomer, green) and H-300 (against the N-terminal region of filaggrin, red) in addition to Hoechst 33258 staining (blue). **c**, Representative histological staining. Green arrowheads show nuclear remnants. **d**, Representative transmission electron microscopy (TEM) images. The red rectangles in the top and the middle panels indicate the regions of the middle and the lower panels, respectively. Red arrowheads show normal corneodesmosomes, and yellow arrowheads show abnormal corneodesmosomes. **e**, Representative three-dimensional immunostaining against ZO-1 (green) and CDSN (red) in addition to Hoechst 33258 staining (blue) in the uppermost layer. The left and right panels show merged and CDSN images, respectively. **f**, Quantitative PCR analysis of FLG2, LOR, CDSN, and ABCA12 expression. The mRNA levels corresponding to the control were set to 100. All the experimental points were obtained from at least 7 independent experiments. Wilcoxon rank sum test: $p = 0.0093$ (FLG2), $p = 0.015$ (LOR), $p = 0.23$ (CDSN), $p = 0.38$ (ABCA12). Scale bar, 50 μm (**b**), 20 μm (**c**, **e**), 4 μm (upper panels in **d**), 1 μm (middle panels in **d**), or 400 nm (lower panels in **d**). p values, * < 0.05, ** < 0.01, *** < 0.001.

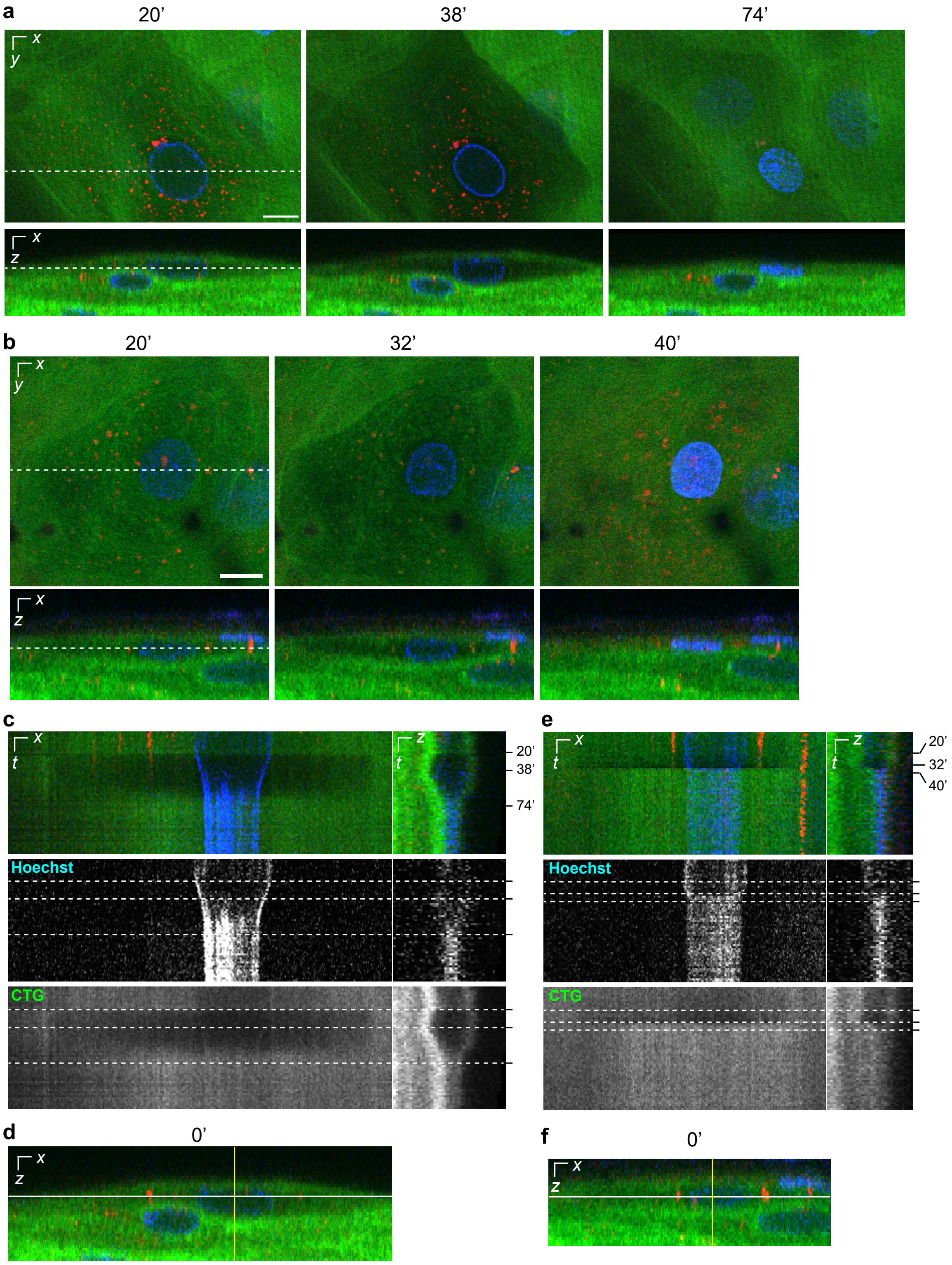

Supplementary Figure 5

The duration of morphological change in the FLG-KD model. **a**, Time-lapse images of the FLG-KD model labelled with Hoechst 33258 (blue), CTG (green), and LTR (red) collected with a 2-min interval. Dashed lines indicate sectional planes. The granular cell shape began to change at 20 min, following which the cell expanded until 38 min and became flattened at approximately 74 min. **b**, Time-lapse images of the control model labelled with Hoechst 33258 (blue), CTG (green), and LTR (red) collected with a 2-min interval. Dashed lines indicate sectional planes. The granular cell shape began to change at 20 min, following which the cells expanded until 32 min and became flattened at approximately 40 min. **c**, Kymographs of the nucleus and cell shape taken from time-lapse images in **a**. Kymographs on the left and right were generated from the white and yellow lines in **d**, respectively. The upper, middle, and lower panels show the merged images, nuclear signals, and CTG signals, respectively. Dashed lines show the time at 20, 38, and 74 min. **d**, An orthogonal image in **a** at 0 min. **e**, Kymographs of the nucleus and cell shape taken from time-lapse images in **b**. Kymographs on the left and right were generated from the white and yellow lines in **f**, respectively. The upper, middle, and lower panels show the merged images, nuclear signals, and CTG signals, respectively. Dashed lines show the time at 20, 32, and 40 min. **f**, An orthogonal image in **b** at 0 min. Scale bar, 10 μm.

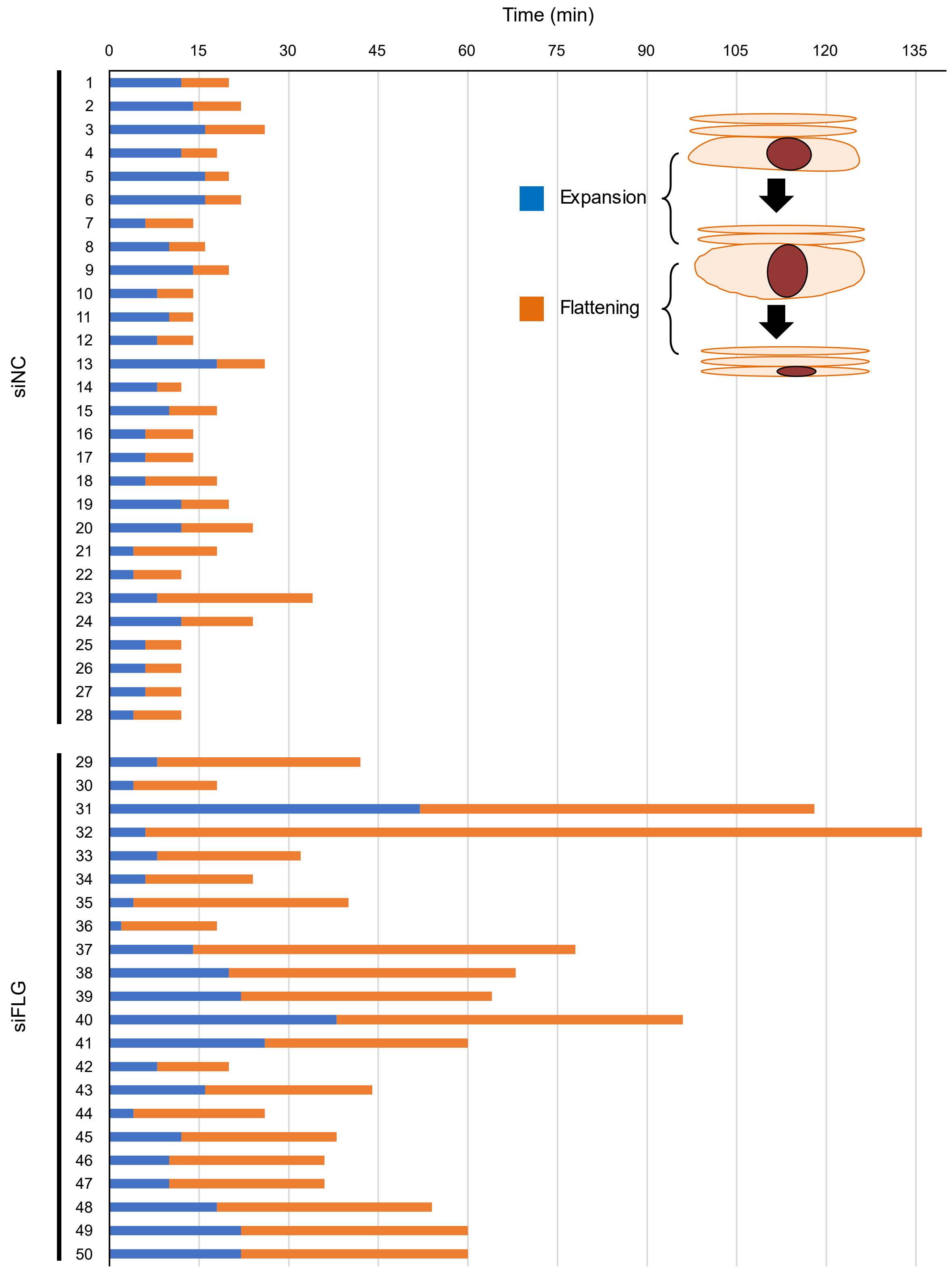

Supplementary Figure 6

A graph of the time from the start of morphological change to expansion (shown as "Expansion") and from expansion to flattening (shown as "Flattening") in individual cells (#1–28, control; #29–50, FLG-KD). The graph corresponds with Fig. 5b.

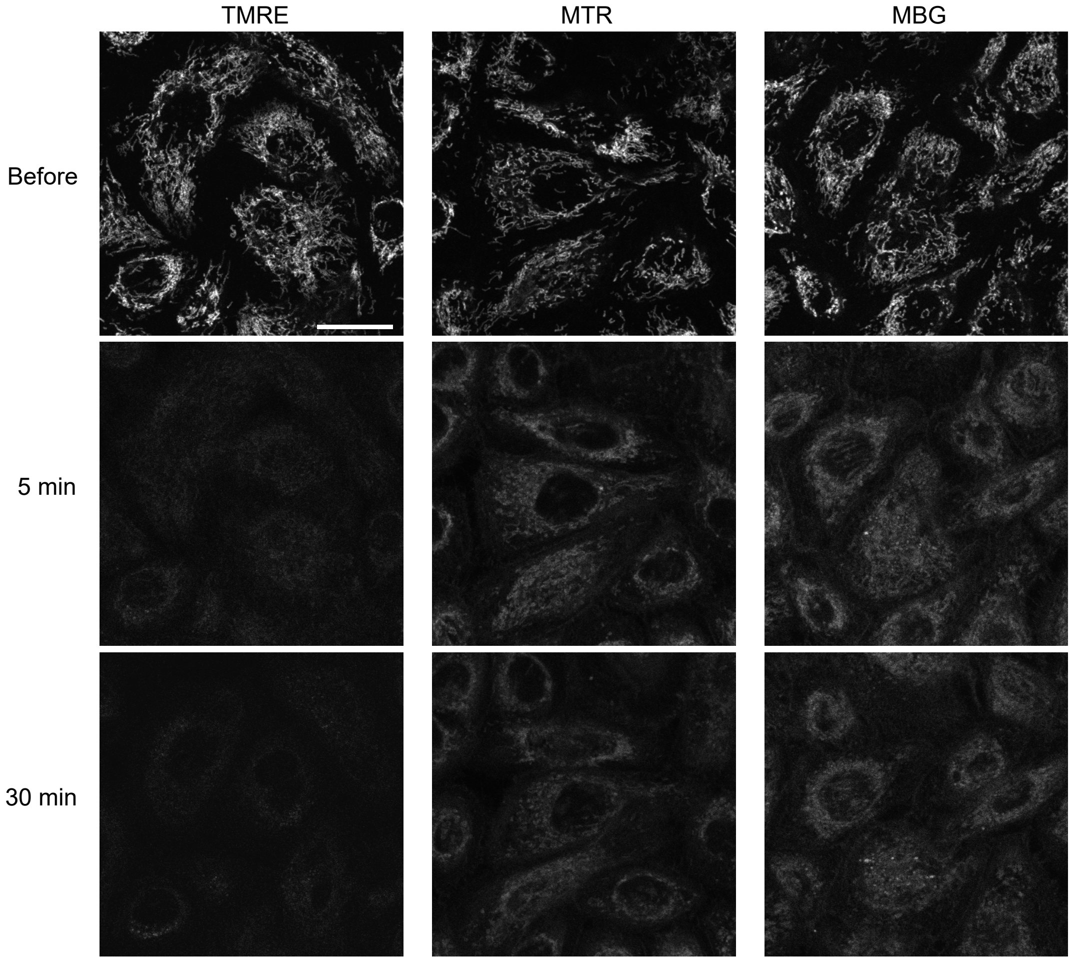

Supplementary Figure 7

Images of keratinocytes labelled with TMRE, MTR, and MBG 5 min and 30 min after treatment with a mixture of antimycin A (10 μM) and oligomycin complex (2 μg/mL) to induce mitochondrial depolarization. The TMRE signals almost completely disappeared after the induction of depolarization, while the MTR and MBG signals were slightly visible. Scale bar, 20 μm.

Supplementary Table 1 Primers and probes used for quantitative PCR.

gene	forward primer	reverse primer	Universal ProbeLibrary probe number
GAPDH	agccacatcgctcagacac	gcccaatacgaccaaatcc	60
FLG	gagaggcggtctgagtcg	gggttcctggagccatgt	32
FLG2	ctgactatggcctgcaacaa	ctttgaccctgaagctttgc	73
LOR	ctcacccttcctggtgctt	ctcacccttcctggtgctt	12
CDSN	atgatggcactgctgctg	aaggtgccaatgctcttagc	51
ABCA12	ctgttaaaccaggcaattcca	aacttgcacaaaagggttcc	50

Supplementary Movie legends

Supplementary Movie 1

Representative movie showing the three-dimensional structure of the epidermal model labelled with Hoechst 33258 (blue) and CTG (green), as shown in Fig. 1a–c. Image size, 160.10 × 160.10 × 63 μm^3.

Supplementary Movie 2

Representative movie showing the morphological change in the epidermal model labelled with Hoechst 33258 (blue) and CTG (green), as shown in Fig. 1c, d. The video plays at 10 frames per second. Scale bar, 10 μm.

Supplementary Movie 3

Representative movie showing DNA leakage from the nuclear region in the epidermal model labelled with Hoechst 33258 (blue) and CTG (green), as shown in Fig. 1f. The video plays at 10 frames per second. Scale bar, 5 μm.

Supplementary Movie 4

Representative movie showing the degradation of DNA in the epidermal model labelled with Hoechst 33258 (blue) and CTG (green), as shown in Fig. 1g. The video plays at 10 frames per second. Scale bar, 20 μm.

Supplementary Movie 5

Representative movie showing the movement of acidic vesicles and morphological change in the epidermal model labelled with Hoechst 33258 (blue), CTG (green), and LTR (red), as shown in Fig. 2d. The video plays at 10 frames per second. Scale bar, 10 μm.

Supplementary Movie 6

Representative movie showing the disappearance of mitochondrial signals in the epidermal model labelled with Hoechst 33258 (blue), CYTO-ID (green), and TMRE (red), as shown in Fig. 4a. The region designated with a white rectangle on the right is magnified and shown on the left. The video plays at 10 frames per second. Scale bar, 10 μm.

Supplementary Movie 7

Representative movie showing the decrease in mitochondrial signals in the epidermal model labelled with Hoechst 33258 (blue), CYTO-ID (green), and MTR (red), as shown in Fig. 4b. The region

designated with a white rectangle on the right is magnified and shown on the left. The video plays at 10 frames per second. Scale bar, 10 μm.

Supplementary Movie 8

Representative movie showing the decrease in mitochondrial signals and stoppage of acidic vesicles in the epidermal model labelled with CTB (blue), MBG (green), and LTR (red), as shown in Fig. 4c. The region designated with a white rectangle on the right is magnified and shown on the left. The video plays at 10 frames per second. Scale bar, 10 μm.

Supplementary Movie 9

Representative movie showing the three-dimensional structure of the FLG-KD model labelled with Hoechst 33258 (blue), CTG (green), and LTR (red). Image size, 228.55 × 228.55 × 52.80 μm^3.

Supplementary Movie 10

Representative movie showing the three-dimensional structure of the control model labelled with Hoechst 33258 (blue), CTG (green), and LTR (red). Image size, 228.55 × 228.55 × 52.20 μm^3.

Supplementary Movie 11

Representative movie showing morphological change in control (left) and FLG-KD (right) models labelled with Hoechst 33258 (blue), CTG (green), and LTR (red), as shown in Supplementary Fig. 5. The video plays at 10 frames per second. Scale bar, 10 μm.
